# Experimental and Numerical Study on Chloride Transport in Unsaturated Concrete: Highlighting Temperature, Humidity, and Mineral Admixtures

**DOI:** 10.3390/ma17040930

**Published:** 2024-02-17

**Authors:** Zhantao Du, Zuquan Jin, Shicai Li, Huan Xue, Rui Zhao

**Affiliations:** 1 School of Civil Engineering, Qingdao University of Technology, Qingdao 266520, China; duzhantao1101@163.com (Z.D.);; 2 Engineering Research Center of Concrete Technology under Marine Environment, Ministry of Education, Qingdao 266520, China; 3 Qingdao Branch of Beijing Zhongchang Engineering Consulting Co., Ltd., Qingdao 266520, China

**Keywords:** unsaturated concrete, chloride transport, diffusion coefficient, numerical simulation, mineral admixtures, temperature–humidity coupling

## Abstract

Chloride transport within concrete is critical for the durability of reinforced concrete structures; however, its diffusion under the coupling action of temperature and humidity has not been fully comprehended. Therefore, in this work, the coupling effects of temperature, relative humidity, and mineral admixtures on chloride transport in concrete were investigated through experimental and numerical simulation work. The results show that the chloride diffusion coefficient decreases with the decreased temperature and growth of relative humidity; however, the chloride concentration on the concrete surface is increased with the growth of temperature and relative humidity. Moreover, compounding about 15% fly ash (FA) and 30% granulated ground blast furnace slag (GGBS) to replace the cement is the most beneficial for improving the antichloride capacity of concrete, considering also the strength. In addition, the numerical simulation considering the coupled effect of temperature and relative humidity of chloride transport in concrete has good agreement with that of experimental results.

## 1. Introduction

Chloride ion is a sensitive keyword when considering the durability performance of reinforced concrete structures, especially under harsh marine environments [1,2,3]. In chloride-rich circumstances, reinforced bars embedded in concrete cover will corrode under the conditions of moisture and oxygen due to the serious damage to the rebars’ electrochemical stability [4,5]. Furthermore, rebar corrosion inside concrete will trigger a series of fatal consequences in the field of civil engineering, which includes the degradation of mechanical properties, load-bearing capacity, and seismic performance of concrete structures. Moisture is not only necessary for cement hydration but is also closely related to the durability of concrete, which plays a role as a carrier for the corrosive media to enter the concrete [6,7]. The moisture variation in concrete is triggered by the water exchange with the external environment. For concrete with a high water–cement ratio, due to its poor compactness, its internal humidity is greatly affected by the environment, and the self-drying effect is not obvious [8,9]. Furthermore, the pore saturation of concrete is determined by the relative humidity; meanwhile, the temperature is considered to influence the diffusion processes. The water transport in concrete not only significantly affects the hardening of concrete but also changes the mass and volume [10,11]. In addition, when the internal relative humidity is higher than the ambient relative humidity, the moisture will be diffused into the air from the concrete inside, resulting in different degrees of dry shrinkage and tensile stress on the surface of the concrete structures, which seriously impacts the service life and durability performance of reinforced concrete structure [12]. Hence, it is of great concern to reveal the principle of chloride ions’ penetration through the concrete cover while evaluating the residual service life of concrete structures [13,14].

Recently, several researchers have conducted extensive studies and attempted to reveal the diffusion process of chloride within concrete using laboratory characterization and numerical simulation [15,16,17,18]. Wu et al. [19] presented a study on the durability testing of a concrete facility located within the North Bay Port region and in service for 200 months. They proposed that the apparent chloride diffusion coefficient (*D*_app_) and the apparent surface chloride concentration (*C*_s_) follow the log-normal distribution, while m follows a normal distribution. Yu et al. [20] proposed the chloride diffusion coefficient model coupled with environmental factors to describe chloride ingress into concrete with field tests and artificially simulated environment experiments. Wang et al. [21] investigated chloride ion transport in concrete, considering the effect of a dry-exposure ratio under a diurnal tidal environment, and established a time-dependent model of chloride ion transport in concrete considering the effect of dry-exposure ratio. Al-Sodani et al. [22] investigated the correlation between short-term chloride ion migration coefficients and long-term Dapp in concrete with indoor experiments and field exposure experiments. They established a correlation model between field and laboratory results based on the experimental data. Pansera et al. [23] proposed an empirical chloride transport model contemplating the convection–diffusion zones through two Gaussian–Lorentzian functions using nonlinear regression in chloride profiles obtained from field structures located in different marine aggressive zones exposed to the marine environment for more than 40 years.

Lately, much mesoscopic research into chloride transport in concrete has been reported [24,25,26,27,28]. These works concentrate on two aspects: (1) investigating the impact of concrete’s mesoscopic phases on chloride transport and (2) developing the equivalent methods from the mesoscale to the macroscale. Lee et al. [29] proposed a coupled transport model considering moisture and chloride distribution in concrete to investigate the influence of a cyclic exposure condition on chloride penetration. Jain et al. [30] developed a numerical model to study the coupled transport of chloride with heat, relative humidity, and oxygen into the concrete. Liu et al. [31] established a modified chloride ion diffusion–convection model for concrete considering curing age, initial water saturation, and the combined effects of chloride ions. These studies have revealed some intrinsic mechanisms of chloride transport in concrete and have demonstrated that mesoscopic research is an effective and reliable method for chloride transport in concrete. However, these investigations did not adequately account for the coupling effects of temperature, relative humidity, and mineral admixtures.

Recently, the authors also conducted relevant studies on this issue, especially in practical marine environments. Despite enormous studies on the point of chloride transportation, there are still several drawbacks to revealing the chloride transport law influenced by other impact factors, such as temperature and relative humidity. Furthermore, a gap still exists in revealing the mechanism of chloride ion transport impacted by external factors. Thus, the chloride ion profile at the concrete cover will be discussed in this paper; meanwhile, concrete with mineral admixture was also involved in this study.

## 2. Experiments

### 2.1. Materials

Ordinary Portland cement (OPC, P·O 52.5), granulated ground blast furnace slag (GGBS, S95), and fly ash (FA, Class I) are used as main cementitious materials. Their chemical compositions are shown in Table 1. Crushed granite with a size of 5–20 mm from Lei Xin Group Company (Qingdao, China) is used as coarse aggregate. River sand with a fineness modulus of 2.6 from Qingjian New Material Company (Qingdao, China) is used as fine aggregate. Polycarboxylic acid superplasticizer (JM-PCA) is used to adjust the workability of fresh concrete. In addition, an air-entraining agent is used to control the air content of fresh concrete by 3–5%.

### 2.2. Experimental Methods

#### 2.2.1. Sample Preparation

The water–binder ratio of concrete was controlled to 0.35. Cubic concrete with the dimensions of 100 mm × 100 mm × 100 mm was prepared as the mix proportion listed in Table 2. Each sample of three is a group and takes the average value as the experimental result. The prepared concrete was cured for 3 d, 7 d, 28 d, 56 d, and 120 d in a standard room with a temperature of 20 ± 2 °C and relative humidity of 95%.

#### 2.2.2. Sensors Arrangement and Monitoring

**Figure 1:**
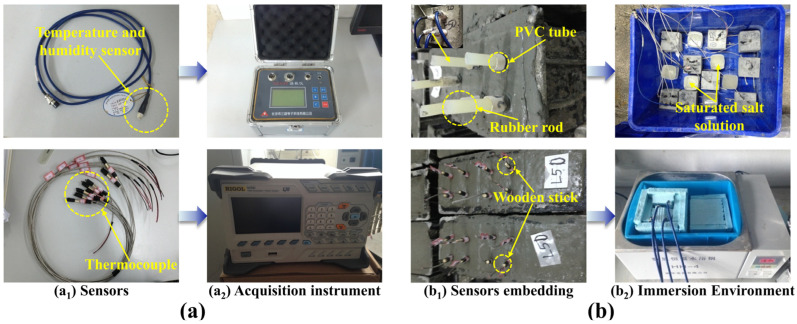
Sensors arrangement and monitoring: (**a**) sensors and their acquisition instrument and (**b**) sensors embedding and immersion environment construction.

The sensors (temperature and humidity sensor and thermocouples) and their acquisition instruments used in this study are presented in Figure 1a. The sensor of temperature and humidity (SZWS-SHT15) is produced by Sanzhi Electronic Technology Co., Ltd. (Changsha, China), and its parameters are as follows: Capacitive, the measured temperature range is 0–120 °C (±0.5 °C), and the measured humidity range is 0–100% (±3%). The thermocouple is K-type and its acquisition instrument (34970A) is produced by Agilent Technology Co., Ltd. (Santa Clara, CA, USA).

The thermocouple was first preprocessed by tying a wooden stick to the bottom of the thermocouple probe to ensure that it could be fixed vertically inside the concrete. After that, a PVC pipe with an inner diameter of 15 mm was inserted into the concrete to a certain depth when pouring into concrete. To prevent the mortar from flowing into the pipe, we placed a rubber rod slightly smaller than the inner diameter of the PVC pipe in advance; after the concrete was finally hardened, the rubber rod was pulled out and replaced by the sensors, as shown in Figure 1b_1_. In this study, four different humidity spaces were constructed as follows: the concrete specimens were immersed in the water to make it fully saturated and reach 100%, as shown in Figure 1b_2_. Then, several specimens were removed in a constant temperature and humidity laboratory with a relative humidity of 80% and a temperature of 20 °C to make the humidity reach 80%. After that, they were dried using a drying oven until the humidity reached 65% and saturated the LiBr solution to make it 45%, respectively. Four temperature conditions of 45%, 65%, 80%, and 100% were constructed, respectively, in four different temperatures (5 °C, 25 °C, 45 °C, and 65 °C); after the salt absorption test, the concrete specimens were cleaned on the surface and then grounded using a type DRB-H1 concrete grinder. Furthermore, the content of chloride ions in powder was evaluated using the electrode method according to the Chinese Testing Code of Concrete for Port and Water Engineering, while saturated potassium sulfate was set as the reference electrode.

## 3. Numerical Simulation

### 3.1. Theoretical Models

**Figure 2:**
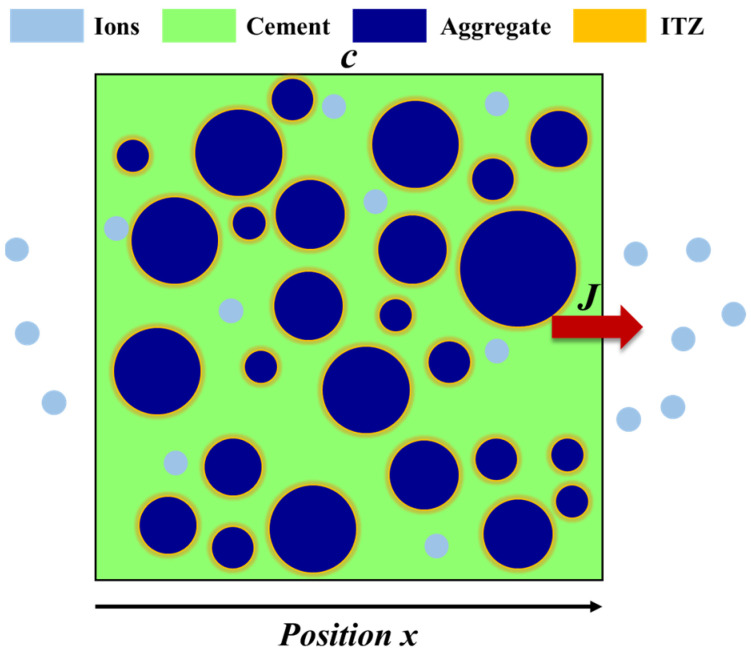
Schematic diagram of ion transport.

The transport of chloride ions in concrete is an unsteady process. Based on Fick’s second law, the transport equation can be expressed as Equation (1) [32]. Figure 2 shows the schematic diagram of ion diffusion within the concrete. Equation (2) is adopted to describe the chloride diffusion process under constant pore saturation. Equation (3) shows the formula to describe the effect of relative humidity on the chloride ion diffusion coefficient [33,34]. Moreover, the chloride migration coefficient will be impacted by temperature, which is presented in Equation (4) [35,36]. The influence of external environmental conditions on chloride ion transport is not a single effect; meanwhile, the increase in temperature will enhance the molecular kinetic energy, thus improving the transport rate of water molecules and resulting in the growth of water in pore structures. Correspondingly, the increase in relative humidity inside the concrete will affect the transport speed of ions or molecules rate, which has a certain effect on the temperature. Equation (5) introduces the empirical formula of chloride ion transport considering the coupled effect of temperature and humidity, while 10 mm was set as a boundary condition [37].

(1) $$\frac{\partial c}{\partial t}=\nabla \left(D\nabla C\right)$$

(2) $$C_{f}=C_{S}\left[1-erf\left(\frac{x}{2\sqrt{Dt}}\right)\right]$$

where *c* is the ions, *t* represents the transport time, *x* represents the transport distance away from the concrete surface, *D* is the migration coefficient, *n*_0_ is a constant, *erf* is the error function, *C_s_* is the surface chloride concentration of concrete, and *C_f_* is the chloride concertation at a certain depth.

(3) $$D={\left[1+\frac{{\left(1-H_{R}\right)}^{4}}{{\left(1+H_{C}\right)}^{4}}\right]}^{-1}$$

(4) D=[TT1]e[1T−1T1]q

(5) {D=D1[TT1]e[1T−1T1]q·[1−7.032(TT1)−0.033×ln(HR)](x<10 mm)D=D1[TT1]e[1T−1T1]q·[1+(1−HR)4(1+HC)4]−1(x≥10 mm)

where *D* is the chloride ion transport coefficient, *H_R_* is the relative humidity inside the concrete, *H_C_* is the critical relative humidity, *T*_1_ is the reference temperature (293 K), *T* is the calculated temperature, *q* is the activation constant, *D*_1_ is the chloride ion transport coefficient at temperature *T*_1_.

### 3.2. Model Establishment

**Figure 3:**
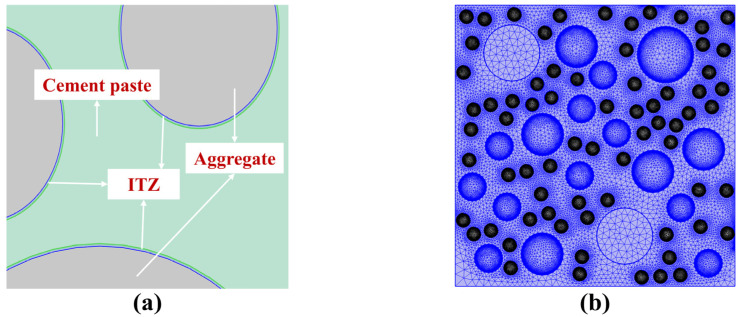
Two-dimensional model of concrete: (**a**) ITZ establishment and (**b**) model meshing.

Based on the theoretical models mentioned above, chloride ion transport was simulated using the software COMSOL Multiphysics (6.1). A two-dimensional model of concrete was established to a cross-section sized 100 mm × 100 mm by randomly distributing circles. The model was divided into three phases: cement paste, fine aggregate, and coarse aggregate. Moreover, the interfacial transitional zone (ITZ) between cement paste and aggregates was modeled with a thickness of 70 μm [38], as shown in Figure 3a. The model was meshed as follows: regular-size triangular meshing for the cement paste, boundary-layer meshing for the interface zone, and finer-size triangular meshing for the aggregates, as shown in Figure 3b. From the physical viewpoint, this model optimizes the aggregate interface and is more in line with the practical situation with a random arrangement of coarse aggregates and fine aggregates. The diffusion coefficient of the ITZ is 4 × 10^−10^ m^2^/s, and that of aggregate is 4 × 10^−13^ m^2^/s. Furthermore, the water–cement ratio (*w*/*c*) was set as 0.35, and the concrete density was 2500 kg/m^3^. In addition, the surface chloride ion concentration can be calculated as Equation (6). The values of the parameters used in the calculations are shown in Table 3.

(6) $$C_{S,Cl}=A_{S,Cl}\left(\frac{w}{b}\right)r_{C,Cl}$$

where *C_S_*_,*Cl*_ is the surface chloride ion concentration, *A_S_*_,*Cl*_ is the regression coefficient of the surface chloride content, *w*/*b* is the water–cement ratio, and *γ_C_*_,*Cl*_ is the fractional coefficient of the surface chloride ion concentration.

## 4. Results and Discussion

### 4.1. Compressive Strength of Concrete

**Figure 4:**
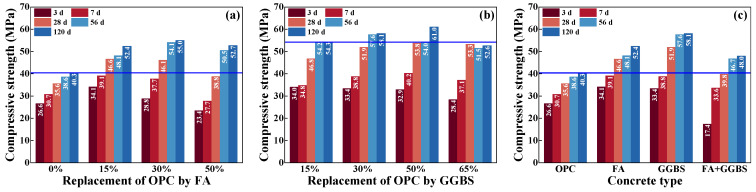
Compressive strength of concrete. (**a**) Effect of FA; (**b**) effect of GGBS; (**c**) effect of compounding FA and GGBS.

The compressive strength of concrete was tested after curing for 3 d, 7 d, 28 d, 56 d, and 120 d, as shown in Figure 4. The early compressive strength (–7 d) of OPC-concrete was improved by the replacement of FA with a dosage of 15%, while it was decreased when the dosage reached 30% and 50%, as shown in Figure 4a. However, the compressive strength of all FA concretes was increased by about 32.4% more than OPC concrete when the curing age reached 120 d. Therefore, the early compressive strength of concrete can be decreased due to the excessive addition of FA, while its effect was positive for its long-term compressive strength. As presented in Figure 4b, the compressive strength of OPC concrete after curing for 28 d was improved by about 31.5%, 45.8%, 51.1%, and 49.7% when the dosage of GGBS was 15%, 30%, 50%, and 65%, respectively. The long-term compressive strength was improved as the same trend. Therefore, the compressive strength of concrete can be improved with the increased dosage of GGBS until the dosage exceeds 50%. In addition, there is no significant improvement in the early compressive strength of OPC concrete when simultaneously adding FA and GGBS, as presented in Figure 4c. Meanwhile, compared to the single addition of FA or GGBS, the compressive strength of FA+GGBS concrete was decreased by about 14.6% and 23.3% for the age of 28 d, and 8.4% and 17.4% for the age of 120 d. Therefore, the compressive strength of concrete can be significantly improved by adding 15% FA or 50% GGBS.

### 4.2. Chloride Transport in Unsaturated Concrete

#### 4.2.1. Effect of Temperature on Chloride Transport

**Figure 5:**
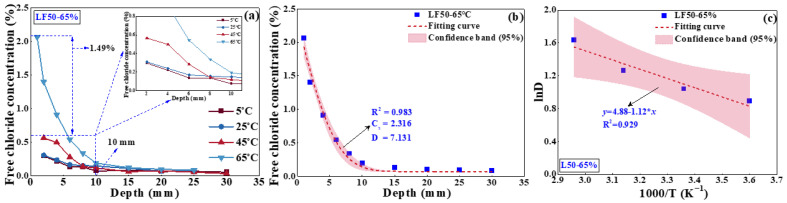
Free chloride concentration of concrete surface: (**a**) effect of temperature, (**b**) fitting curve, and (**c**) regression results between chloride diffusion coefficient and temperature.

The free chloride concentration of LF50 concrete surface was measured after eroding in the environment with an RH of 65% and four temperatures (5 °C, 25 °C, 45 °C, and 65 °C) for 10 d, as presented in Figure 5. The chloride concentration on the surface area was 0.290% at 5 °C, which tends to be stable at a depth of approximately 5 mm. Similarly, at 25 °C, the chloride concentration at the surface was about 0.275%, and the diffusion depth was about 5 mm. However, the surficial chloride concentration was significantly improved to 0.610% and 2.264% when the temperature reached 45 °C and 65 °C, respectively. Meanwhile, the diffusion depth was also increased to 10 mm. The chloride ion distribution after capillary salt absorption at 65 °C was fitted according to Fick’s second law to obtain the diffusion coefficient (*D*) and chloride concentration (*C_s_*) of the concrete surface, as shown in Figure 5b. The correlation coefficient (*R*^2^) was 0.983, indicating the fitting result was reliable. The confidence band of 95% presented in the figure can also prove that result. In addition, the correlation between the chloride diffusion coefficient and temperature was established according to Arrhenius’ empirical formula (Equation (7)), as presented in Figure 5c. The chloride diffusion coefficient of concrete gradually increased with the increased temperature; meanwhile, the migration rate of chloride showed a similar tendency. From a physical point of view, the chloride transport was accelerated when the temperature increased; meanwhile, the concentration was higher due to a faster chloride migration coefficient.

(7) $$\frac{d\ln D}{dT}=\frac{E_{a}}{RT^{2}}$$

where the slope is the apparent activation energy *E_a_* (j·mul^−1^), the intercept is the preexponential factor, *A*, *D* is the chloride diffusion coefficient (10^−12^ m^2^/s), and *T* is the thermodynamic temperature (*K*).

#### 4.2.2. Effect of Relative Humidity on Chloride Transport

**Figure 6:**
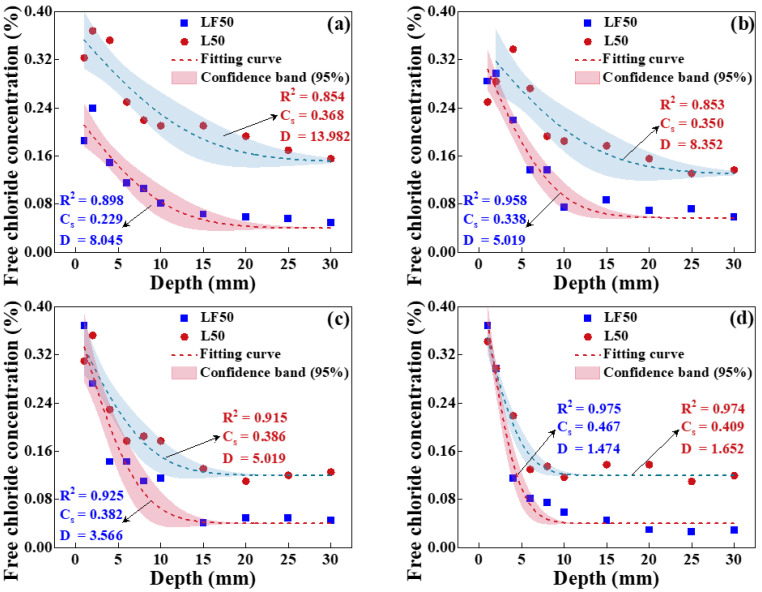
Free chloride concentration of LF50 concrete at the relative humidity of (**a**) 45%, (**b**) 65%, (**c**) 80%, and (**d**) 100%.

The free chloride concentration of concrete (L50 and LF50) surface was measured after eroding in an environment with a temperature of 25 °C and four relative humidities (45%, 65%, 80%, and 100%) for 10 d, as presented in Figure 6. Meanwhile, the diffusion coefficient and chloride concentration of the concrete surface obtained by fitting according to Fick’s second law. The diffusion coefficient of concrete is decreased with the increased relative humidity. However, the *C_s_* is increased with the growth of relative humidity, especially for LF50 concrete. The *C_s_* of LF50 concrete is increased from 0.229% to 0.467% when the relative humidity is increased from 45% to 100%. On the one hand, the chloride ion diffusion is inhibited by the compound replacement of FA and GGBS, inducing more chlorides to be enriched on the concrete surface. Moreover, chloride diffusion is dependent on moisture transport, while more moisture migrates from the concrete surface to the interior when the relative humidity of the environment is lower. It can be further observed that the penetration depth of concrete decreases with the increase in internal humidity; the penetration depth of chlorides is about 20 mm, 15 mm, 15 mm, and 10 mm when the relative humidity is 45%, 65%, 80%, and 100%. In addition, it is noted that under unsaturated conditions (not a relative humidity of 100%), there are convection zones that exist on the concrete surface due to capillary adsorption. Therefore, the chloride diffusion will be inhibited by the increased relative humidity; meanwhile, the chloride will be enriched on the concrete surface.

**Figure 7:**
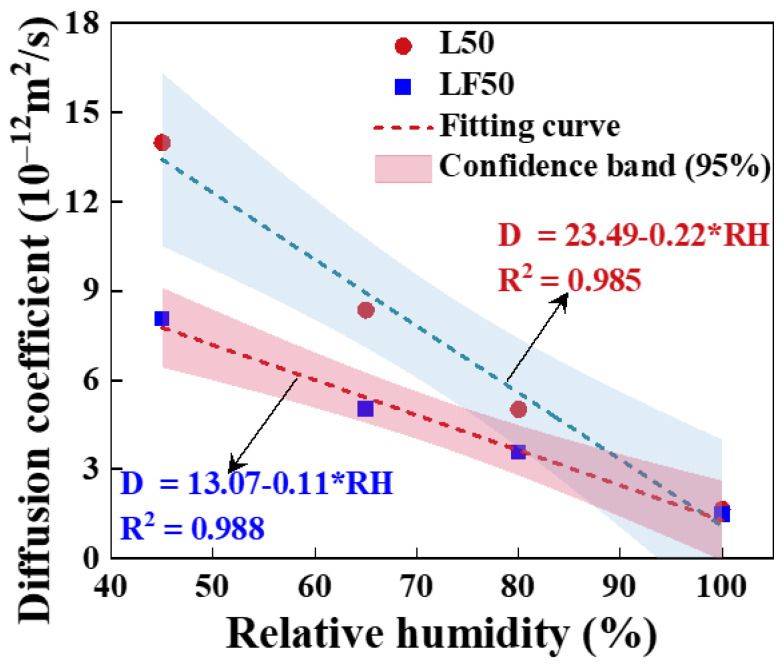
The fitting result of the chloride diffusion coefficient and relative humidity.

Figure 7 shows the fitting result of the chloride diffusion coefficient and relative humidity in concrete. A new function is established, as shown in Equation (8). There is a linear decreasing trend of the chloride diffusion coefficient with the rising of the relative humidity. The main reason for this phenomenon is that the capacity of chloride penetration is stronger when relative humidity is low due to the water absorption ability. Meanwhile, chloride ion concentration will be much higher, accompanied by moisture going inside the concrete. The capillary absorption capacity is weakened and almost zero when the relative humidity reaches 100%.

(8) $$D=a-bR_{H}$$

where *D* is the chloride migration coefficient (10^−12^ m^2^/s), *R_H_* is the relative internal humidity of concrete, *a* and *b* are constant values which depend on the concrete type and temperature.

#### 4.2.3. Effect of Mineral Admixtures on Chloride Transport

(1) Effect of adding FA on chloride transport

**Figure 8:**
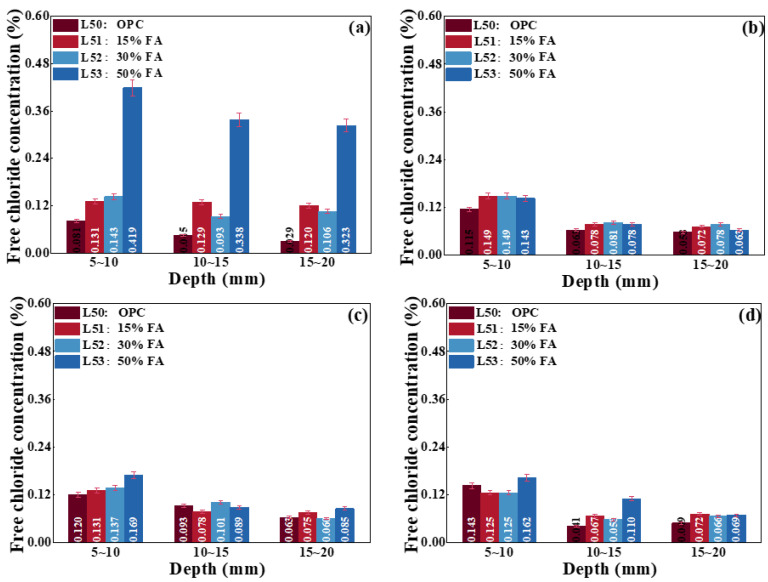
Chloride ion concentration of concrete with different contents of FA under the relative humidity of (**a**) 45%, (**b**) 65%, (**c**) 80%, and (**d**) 100%.

Figure 8 shows the chloride ion concentration of concrete with different contents of FA under different internal relative humidities. There is no significant variation in chloride ion content at the same depth, especially for the concrete prepared with 15% and 30% FA. However, for the concrete in the environment with a relative humidity of 45%, its chloride ion concentration is increased significantly at the same depth when the replacement of FA reaches 50%. Therefore, there is little effect on porosity and chloride diffusion coefficient when the content of fly ash is less than 30%. In addition, FA is advanced in larger surface area and hollow structure, which provides the reaction sites and increases the absorption capacity of chloride ions. Therefore, considering the strength and chloride diffusion of concrete, the replacement of FA is suggested as less than 30%.

(2) Effect of adding GGBS on chloride transport

**Figure 9:**
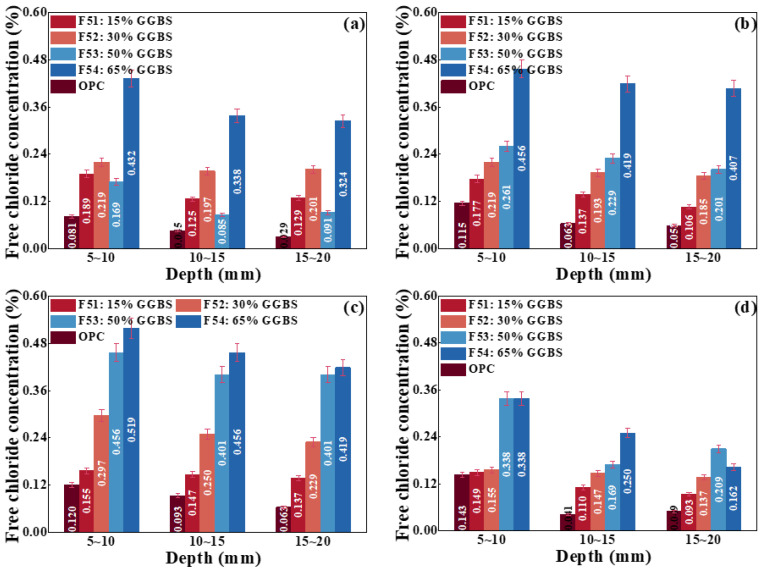
Chloride ion concentration of concrete with different contents of GGBS under the relative humidity of (**a**) 45%, (**b**) 65%, (**c**) 80%, and (**d**) 100%.

Figure 9 shows the chloride ion concentration of concrete with different contents of GGBS under different relative humidities. For F53 concrete under the relative humidity of 45%, its chloride ion concentration is the lowest at the same depth, indicating that the concrete with 50% GGBS admixture has the strongest antichloride capacity. For the concrete under the relative humidity of 45%, 65%, and 80%, respectively, the chloride ion concentration at the same depth is gradually increased, which is higher than that of the concrete without GGBS mixing. Therefore, considering the strength and chloride diffusion of concrete, the replacement of GGBS is suggested as less than 50%.

(3) Effect of compounding FA and GGBS on chloride transport

**Figure 10:**
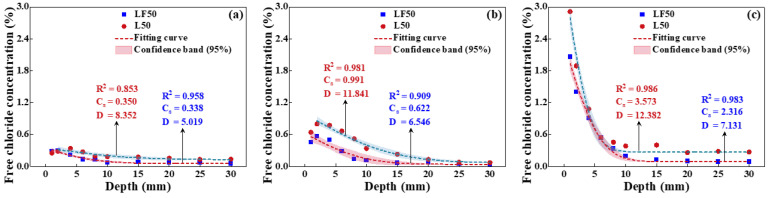
Chloride ion concentration in concretes under the temperatures of (**a**) 25 °C, (**b**) 45 °C, and (**c**) 65 °C.

Figure 10 presents the evolution of chloride ion concentration in concretes under the relative humidity of 65% and different temperatures. The chloride diffusion is inhibited by compounding FA and GGBS, especially under higher temperatures. Meanwhile, the difference in the chloride diffusion coefficient between the normal concrete and that of compounding FA and GGBS is more significant with the increased temperature. The chloride diffusion coefficient of LF50 concrete under the temperature of 65 °C is decreased by about 42.4% less than that of L50 concrete. Therefore, compounding 17% FA and 32% GGBS to replace the OPC is beneficial for improving the antichloride capacity of concrete. Meanwhile, the strength and porosity of concrete is also improved.

In summary, temperature, relative humidity, and mineral admixtures will influence the chloride ion transport at the concrete surface. Yet, only individual effects of temperature, relative humidity, and mineral admixtures on the chloride ion transport were analyzed in this paper; also, the conditions of impact factors were limited in this experiment, and a gap with the reality in practical engineering still exists, which may influence the service life prediction of reinforced concrete structures. Moreover, the coupling effects have not been investigated in detail and still need to be deeply studied in the not-distant future.

### 4.3. Numerical Simulation

#### 4.3.1. Single Effect of Temperature or Relative Humidity on Chloride Transport

(1) Effect of temperature on chloride transport

**Figure 11:**
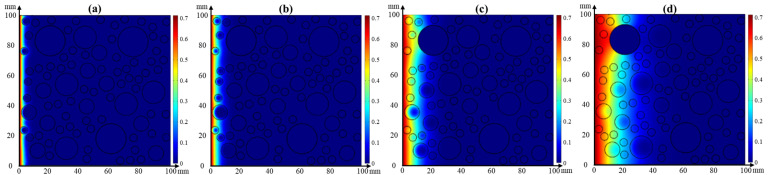
Cloud images of chloride distribution in concrete at the temperature of (**a**) 5 °C, (**b**) 25 °C, (**c**) 45 °C, and (**d**) 65 °C.

Based on Equation (4), the single effect of temperature on chloride transport in concrete for 30 d was simulated using the COMSOL software. Figure 11 presents the cloud images of chloride distribution in concrete at the temperatures of 5 °C, 25 °C, 45 °C, and 65 °C. The chloride concentration is decreased with the depth of concrete, and the chloride migrates to greater depths with the increase in temperature. The chloride diffusion depth in concrete is 5 mm, 7.5 mm, 12.5 mm, and 25 mm when the temperature is 5 °C, 25 °C, 45 °C, and 65 °C, respectively. Compared to the experimental result, the effect tendency of temperature on chloride transport obtained with simulation is identical. However, it should be noted that the effect of relative humidity is not considered in Equation (4), inducing an error existing in the diffusion depth. In addition, the chloride enrichment at the ITZ between the aggregate and cement paste is weakened when the temperature reaches 65 °C.

(2) Effect of relative humidity on chloride transport

**Figure 12:**
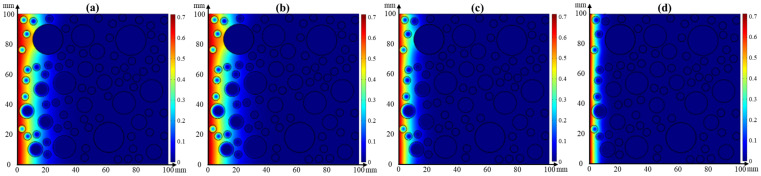
Cloud images of chloride distribution in concrete with a relative humidity of (**a**) 45%, (**b**) 65%, (**c**) 80%, and (**d**) 100%.

Based on Equation (3), the single effect of relative humidity on chloride transport in concrete for 30 d was simulated using the COMSOL software. Figure 12 presents the cloud images of chloride distribution in concrete at the relative humidity of 45%, 65%, 80%, and 100%. The chloride concentration is decreased with the depth of concrete, and the chloride migrates to greater depths as relative humidity decreases. This effect tendency of relative humidity on chloride transport is consistent with the experimental result presented in Figure 6. The chloride diffusion depth in concrete is 25 mm, 20 mm, 12.5 mm, and 7.5 mm when the relative humidity is 45%, 65%, 80%, and 100%, respectively. In addition, the chloride is enriched at the ITZ between the aggregate and cement paste, and this phenomenon becomes more pronounced with the decrease in relative humidity. The main reason is that the moisture transport is inhibited by the aggregate, resulting in the decrease in chloride diffusion to the interior of the aggregate.

#### 4.3.2. Coupled Effect of Temperature and Relative Humidity on Chloride Transport

**Figure 13:**
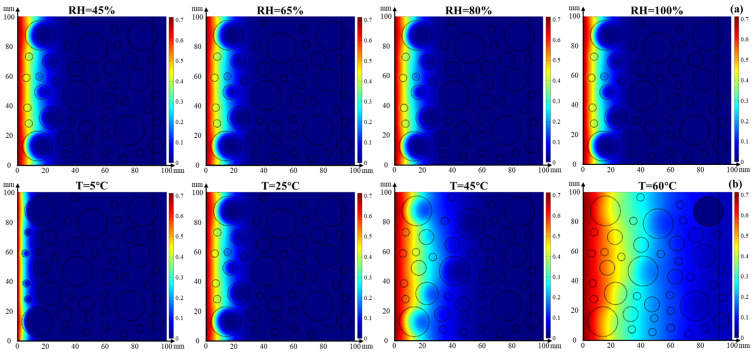
Cloud images of chloride distribution in concrete at (**a**) constant temperature of 25 °C and (**b**) constant relative humidity of 45%.

Based on Equation (5), the coupled effect of temperature and relative humidity on chloride transport in concrete for 30 d was simulated using the COMSOL software. Figure 13 presents the cloud images of chloride distribution in concrete at a constant temperature and relative humidity. The chloride concentration is decreased with the depth of concrete. The effect of temperature on chloride transport in concrete is more significant than that of relative humidity because the chloride migrates to greater depths as the temperature, as shown in Figure 13b. The chloride migrates to the deepest interior of the concrete when the relative humidity is 45% and the temperature is 25 °C. However, there is no significant difference in the chloride transport in concrete at the constant temperature and different relative humidity. It is worth noting that the chloride diffusion capacity of coarse aggregate is improved by the higher temperature while the transport distance is prolonged; furthermore, coarse aggregate has better resistance to chloride penetration than fine aggregates. In addition, the phenomenon of chloride enrichment in the ITZ between the aggregate and cement paste is alleviated.

## 5. Conclusions

In this work, the coupling effect of temperature, relative humidity, and mineral admixtures (FA and GGBS) on chloride transport in concrete was investigated through experimental and numerical simulation work. The main conclusions are as follows:

1. The replacement of OPC by FA and GGBS is beneficial for improving the strength and capacity of antichloride, and its best dosage is suggested as compounding about 15% FA and 30% GGBS.
2. The chloride diffusion coefficient is decreased with the decreased temperature and growth of relative humidity; however, the chloride concentration on the concrete surface is increased with the growth of temperature and relative humidity. Moreover, the chloride migrates to the deeper interior as the temperature increases.
3. The effect tendency of temperature and relative humidity on chloride distribution in concrete obtained from the numerical simulation is consistent with the experimental result. Moreover, its accuracy is improved when considering the coupled effect of temperature or relative humidity on chloride transport.

## Figures and Tables

**Table 1:** Chemical compositions of binder materials (wt.%).

Constituent	SiO_2_	Al_2_O_3_	Fe_2_O_3_	CaO	MgO	Na_2_O	K_2_O	SO_3_	P_2_O_5_
OPC	21.58	4.95	3.29	64.84	3.48	0.56	0.48	0.53	0.12
GGBS	29.05	12.51	1.30	48.34	5.72	0.56	0.08	1.18	0.54
FA	56.90	13.70	4.46	1.90	0.37	0.12	1.55	0.12	0.11

**Table 2:** Mix proportions of concrete (kg/m^3^).

NO.	Cement	GGBS	FA	Sand	Aggregate	Water	Superplasticizer	Air-Entraining Agent
L50	470	–	–	760	1090	165	8	0.19
L51	399.5	–	70.5	760	1090	165	8	0.25
L52	329	–	141	760	1090	165	8	0.25
L53	235	–	235	760	1090	165	8	0.25
F51	399.5	70.5	–	760	1090	165	8	0.12
F52	329	141	–	760	1090	165	8	0.12
F53	235	235	–	760	1090	165	8	0.12
F54	165	305	–	760	1090	165	8	0.12
LF50	240	150	80	760	1090	165	8	0.25

**Table 3:** Parameters and values for calculation.

Parameter	Value	Units
*D*_1_	4 × 10^−12^	m^2^/s
*D*_2_	4 × 10^−10^	m^2^/s
*D*_3_	4 × 10^−13^	m^2^/s
*T*_ref_	293.15	K

Note: *D*_1_, *D*_2_, and *D*_3_ are the chloride diffusion coefficients of the cement paste, ITZ, and aggregate (coarse and fine aggregate), respectively. *T*_ref_ is the standard temperature. *q* is the activation constant.

## Data Availability

Data are contained within the article.
